# Exercise for frailty research frontiers: a bibliometric analysis and systematic review

**DOI:** 10.3389/fmed.2024.1341336

**Published:** 2024-05-01

**Authors:** Wenyuan Xu, Xianghu Zhao, Meiling Zeng, Shengbing Wu, Yikang He, Meiqi Zhou

**Affiliations:** ^1^Graduate School, Anhui University of Chinese Medicine, Hefei, China; ^2^Department of Rehabilitation, Zhongda Hospital, School of Medicine, Southeast University, Nanjing, China; ^3^College of Sports Medicine, Wuhan Sports University, Wuhan, China; ^4^Normal College, Chengdu University, Chengdu, China; ^5^Institute of Acupuncture and Meridian, Anhui Academy of Chinese Medicine, Hefei, China; ^6^Anhui Province Key Laboratory of Meridian Viscera Correlationship, Hefei, China

**Keywords:** exercise, frailty, old adults, bibliometric analysis, visualization analysis

## Abstract

**Background:**

Exercise intervention is a method of improving and preventing frailty in old age through physical exercise and physical activity. It has a positive impact on many chronic diseases and health risk factors, in particular cardiovascular disease, metabolic disease, osteoporosis, mental health problems and cancer prevention, and exercise therapies can also fight inflammation, increase muscle strength and flexibility, improve immune function, and enhance overall health. This study was aimed to analyze research hotspots and frontiers in exercise therapies for frailty through bibliometric methods.

**Methods:**

In this study, data of publications from 1st January 2003 to 31st August 2023 were gathered from the Web of Science Core Collection and analyzed the hotspots and frontiers of frailty research in terms of remarkable countries/regions, institutions, cited references, authors, cited journals, burst keywords, and high-frequency keywords using CiteSpace 6.2.R3 software. The PRISMA reporting guidelines were used for this study.

**Results:**

A collection of 7,093 publications was obtained, showing an increasing trend each year. BMC Geriatrics led in publications, while Journals of Gerontology Series A-Biological Sciences and Medical Sciences dominated in citations. The United States led in centrality and publications, with the University of Pittsburgh as the most productive institution. Leocadio R had the highest publication ranking, while Fried Lp ranked first among cited authors. Keywords in the domain of exercise therapies for frailty are “frailty,” “older adult,” “physical activity,” “exercise,” and “mortality,” with “sarcopenia” exhibiting the greatest centrality. The keywords formed 19 clusters, namely “#0 older persons,” “#1 mortality,” “#2 muscle strength,” “#3 bone mineral density,” “#4 muscle mass,” “#5 older adults,” “#6 older people,” “#7 women’s health,” “#8 frail elderly,” “#9 heart failure,” “#10 geriatric assessment,” “#11 comprehensive geriatric assessment,” “#12 outcm,” “#13 alzheimers disease,” “#14 quality of life,” “#15 health care,” “#16 oxidative stress,” “#17 physical activity,” and “#18 protein.”

**Conclusion:**

This study presents the latest developments and trends in research on frailty exercise intervention treatments over the past 20 years using CiteSpace visualization software. Through systematic analyses, partners, research hotspots and cutting-edge directions were revealed, providing a guiding basis for future research.

## 1 Introduction

Frailty is a multidimensional, comprehensive concept that involves a decrease in the ability to function in multiple aspects such as physical, psychological, and social, accompanied by an elevated susceptibility to stressors (1, 2). With the increasing aging of the population, the phenomenon of frailty is gaining more and more attention. Frailty has become prevalent among old adults, not only being associated with an increased risk of fractures, recurrent falls, and disability (2), but also posing an elevated risk of adverse outcomes in patients with cardiovascular disease (CVD) (3). Frailty seriously affects the daily life and social participation of old adults to some extent. Therefore, it is important to find and explore economical, convenient and effective methods of frailty prevention and rehabilitation (4). However, physical activity plays a crucial role in preventing and treating frailty, even in the absence of effective pharmacological interventions. Physical activity has been recognized as a key recommendation for managing frailty, as highlighted by its inclusion in the Lancet guidelines for frailty management in 2019 (5). Resistance exercise, aerobic exercise, flexibility exercise, balance function training, traditional Chinese exercise and other auxiliary exercise methods are commonly used. Combining aerobic and resistance exercises with diet-induced weight loss significantly improves physical function and reduces frailty in the old adults with obesity (6). Exercise improves frailty by increasing muscle strength and endurance, regulating the ratio of fast and slow muscle fibers, improving neuromuscular adaptation, promoting bone health and body metabolism, and improving cardiorespiratory fitness.

Therefore, it is important to perform a comprehensive review of exercise intervention studies related to frailty. In this study, the visualization software CiteSpace was used to analyze a total of 7,093 publications associated with exercise therapies for frailty for the period from 1st January 2003 to 31st August 2023. Through visual bibliometrics, the current condition and trends of exercise intervention frailty research were comprehensively presented from multiple perspectives, such as the annual publication count, countries, institutions, keywords, citations and authors. This research is aimed to be a valuable reference for future study on exercise therapies for frailty, providing important insights and guidance.

## 2 Methods

### 2.1 Data sources

In this paper, we chose the Web of Science Core Collection (WoSCC) for data collection. Web of Science has a wide range of disciplinary coverage, high-quality literature resources, powerful retrieval and data analysis, etc. Meanwhile, the citation indexing function of Web of Science allows researchers to track and analyze the citation of the literature, and to understand the citation frequency and citation network of the literature; it also provides indexes such as the impact factor of journals, which can help researchers to assess the academic reputation and influence of the journals. Web of Science is a widely used and recognized academic database, and many academic institutions, researchers and scientific research institutes use Web of Science for bibliometric analysis.

### 2.2 Search strategy

On the basis of the WoSCC, the search strategy was as described: [TS = (“physical activity” OR training OR “resistant training” OR exercise) and (frailty OR frail) and Articles or Review (Document Types) and English (Languages)].

### 2.3 Selection criteria

**Figure 1:**
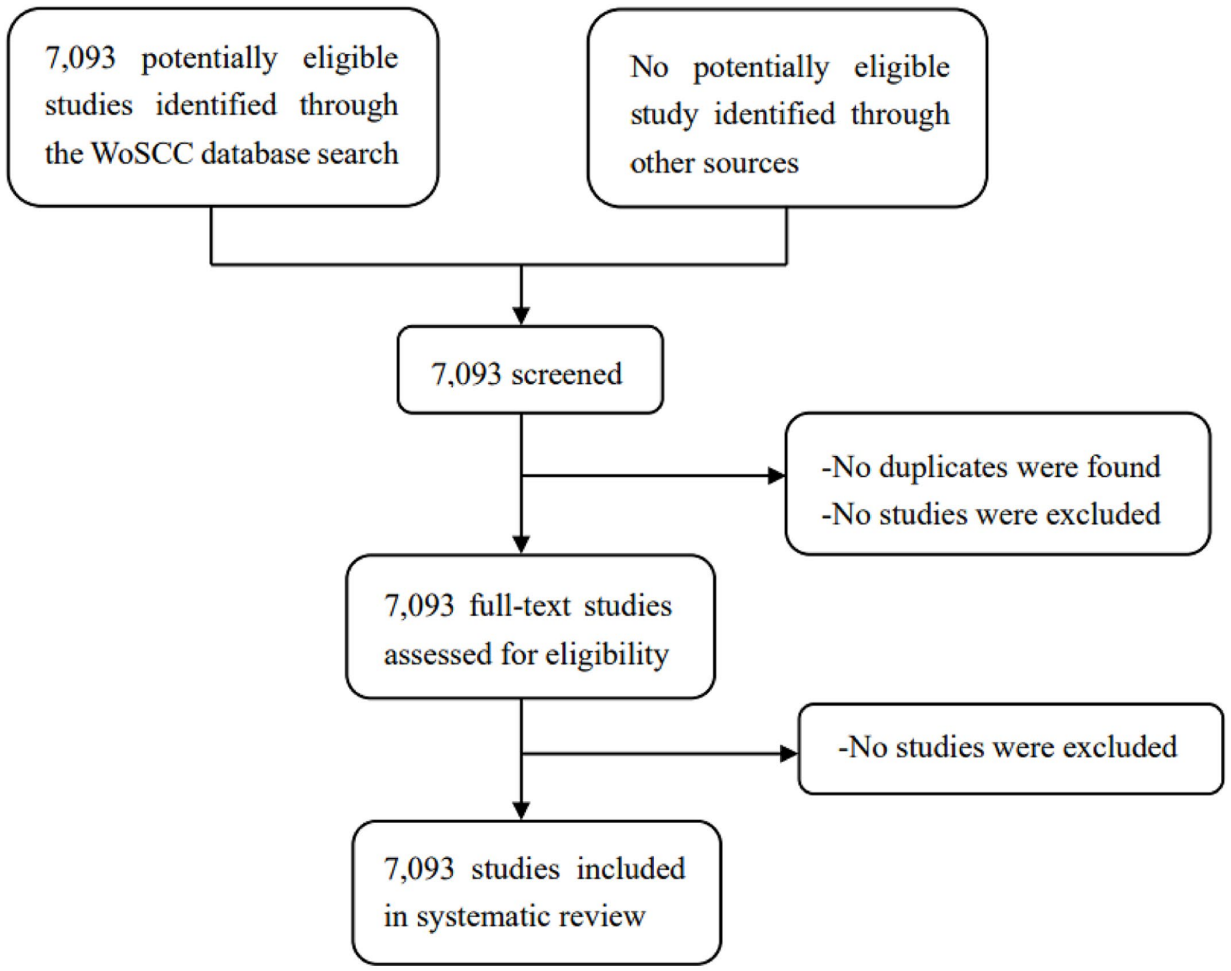
Study selection.

This study included articles and reviews written in English and retrieved from the WoSCC database. The search covered the period from January 1, 2003, to August 31, 2023, resulting in an initial collection of 7,093 records. Two researchers independently assessed the titles and abstracts to exclude records unrelated to exercise therapies for frailty. Then, de-duplication was performed by CiteSpace, and finally all records were included in this study. Following the evaluation of titles and abstracts, 7,093 relevant records were included in this study. Among them, 5,965 articles and 1,128 reviews were included. The PRISMA reporting guidelines were used for this study (Figure 1).

### 2.4 Data analysis

The 7,093 records were further analyzed using CiteSpace software. CiteSpace is a web-based Java application used for analyzing and visualizing data (7). It is a highly regarded and influential software in the field of information visualization analysis, providing valuable insights for research area analysis (8). This study employed an annual time slice, with nodes categorized as country/region, keyword, reference, author, institution, cited author and journal. During the analysis of countries/regions, institutions, authors, and keywords, this study focused on the top 10 levels of citations for each year. This study utilized the g-index to analyze cited references, journals, and authors. The pruning module included Pathfinder, pruning sliced networks and the merged network, with default parameter settings. Co-occurrence and cluster visualizations were utilized to present the findings. CiteSpace was utilized to identify key areas of focus and emerging trends among remarkable countries/regions, institutions, authors, cited references, cited journals, high-frequency keywords, and burst terms. Influence rankings of authors, countries/regions, and institutions were determined based on frequency and centrality. Node sizes and colors in the figures were significant factors.

## 3 Results

### 3.1 Analysis of yearly publication trends

**Figure 2:**
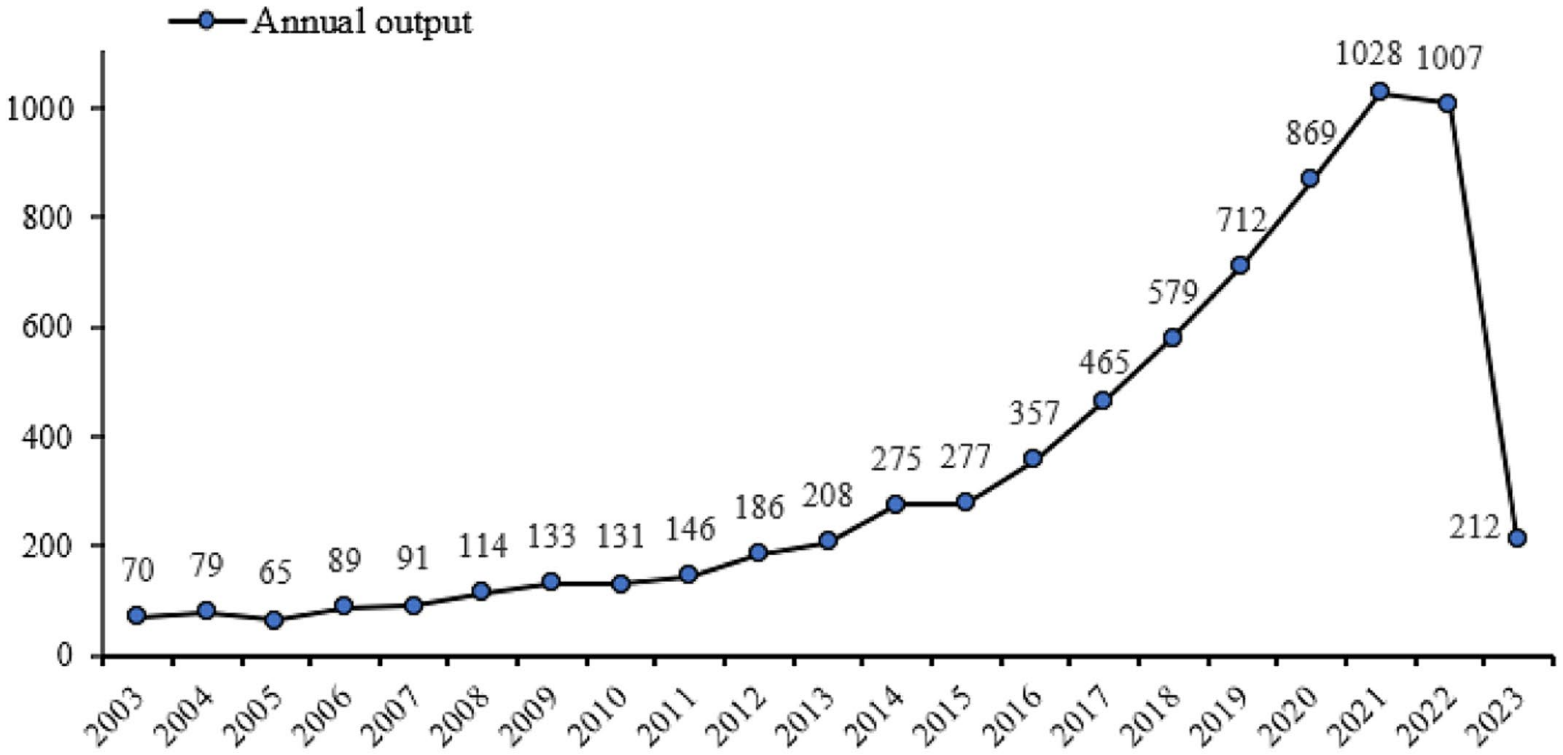
Annual number of published articles from 2003 to 2023.

The WoSCC database includes 7,093 records on exercise therapies for frailty. The line figure illustrates the publication trend over time, showing the number of papers published each year (Figure 2). Based on the shown figure, it is evident that the literature regarding to this subject emerged in 2003, and there has been a consistent annual growth in the number of papers. It should be noted that the data for 2023 only accounts for the period from January to August, but it is anticipated that the upward trend in publications will persist throughout the year. From 2003 to 2015, there was a modest volume of publications; however, the overall development displayed an upward trend, indicating a gradual increase in research interest concerning exercise therapies for frailty during this time. Subsequently, starting from 2015, there was a notable surge in publications, which indicates a heightened research emphasis in this domain. Notably, exercise rehabilitation has emerged as a hot spot in the study of frailty.

### 3.2 Analysis of journals and references

Table 1 presents the top 10 journals in terms of the highest publication count for exercise therapies in frailty studies. Out of these 10 journals, only International Journal of Environmental Research and Public Health is a general journal, the other remaining journals are geriatrics journals. There are four with an IF value of more than 5.0. The top two journals in terms of IF are Journal of the American Medical Directors Association and Journal of the American Geriatrics Society, with values of 7.802 and 7.538, respectively.

**Table 1:** Top 10 most productive journals related to exercise therapies for frailty.

Journal	Publication	IF
BMC Geriatrics	246	4.070
Journal of the American Geriatrics Society	175	7.538
International Journal of Environmental Research and Public Health	174	4.614
Journals of Gerontology Series A Biological Sciences and Medical Sciences	172	6.591
Journal of Nutrition Health Aging	165	5.285
Journal of the American Medical Directors Association	162	7.802
Archives of Gerontology and Geriatrics	142	4.163
Experimental Gerontology	115	4.253
Aging Clinical and Experimental Research	114	4.481
Geriatrics Gerontology International	113	3.387

IF, impact factor; IF in category according to Journal Citation Reports (2021).

**Figure 3:**
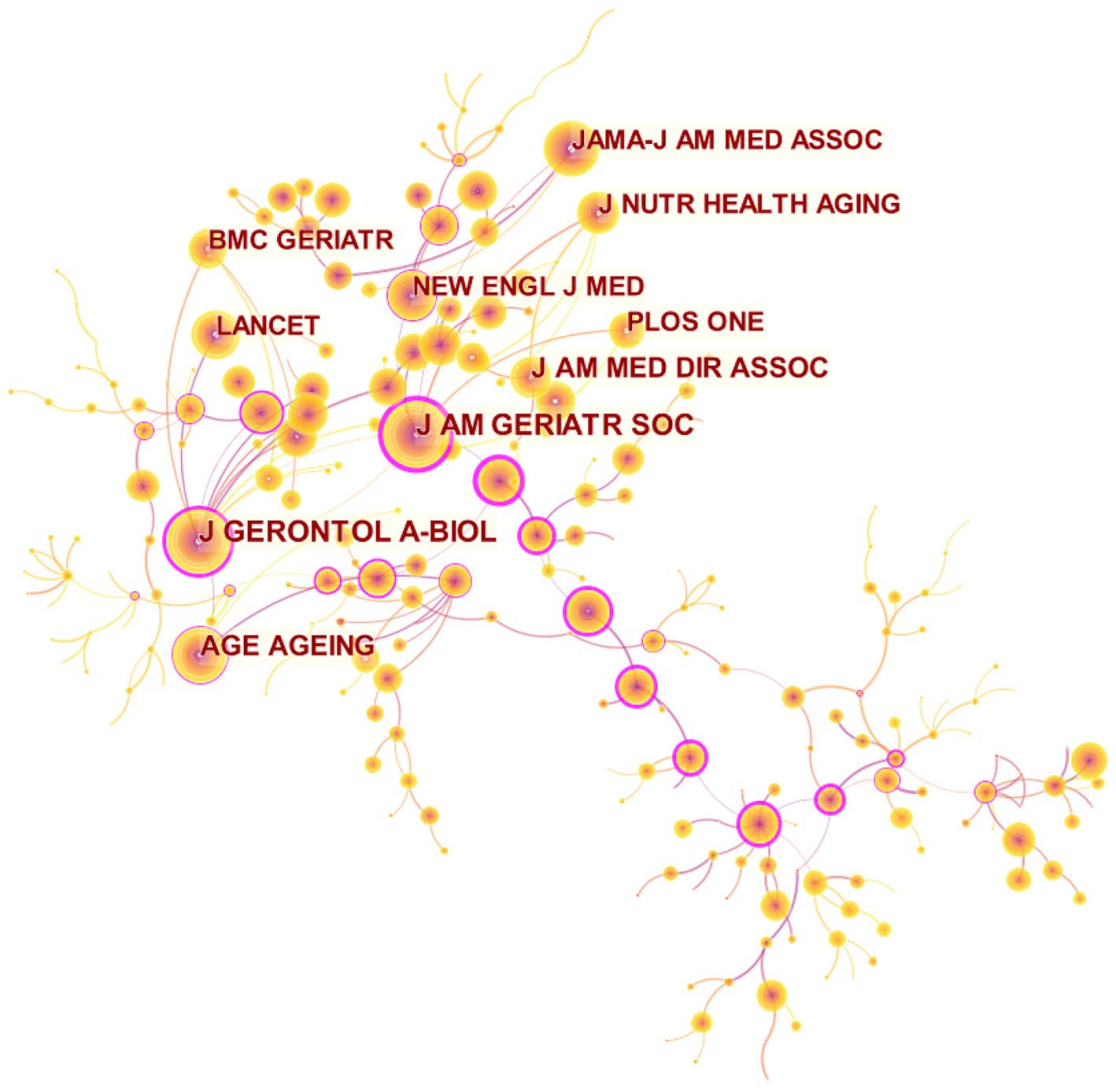
Map of cited journals for exercise interventions for frailty from 2003 to 2023.

Furthermore, Figure 3, generated by CiteSpace, displays the cited journal map consisting of 306 nodes and 259 links. Nodes represent journals, while links show co-citation relationships. Notably, certain nodes present purple rings, indicating their high centrality in the network. Among the top five journals with centrality scores over 0.05, the two most cited journals based on centrality are the Journal of The American Geriatrics Society and Journals of Gerontology Series A-Biological Sciences and Medical Sciences, having centrality scores of 0.73 and 0.53, respectively. Table 2 presents the top 10 journals associated with exercise therapies for frailty.

**Table 2:** Top 10 cited journals related to exercise therapies for frailty.

Cited journal	Frequency	Centrality
Journals of Gerontology Series A-Biological Sciences and Medical Sciences	4,774	0.53
Journal of the American Geriatrics Society	4,694	0.73
Age and aging	3,223	0.16
Journal of the American Medical Directors Association	2,724	0.05
Lancet	2,485	0.01
JAMA-Journal of the American Medical Association	2,460	0.08
Plos One	2,241	0.01
Journal of Nutrition Health & Aging	2,128	0.08
BMC Geriatrics	2,126	0.02
New England Journal of Medicine	1903	0.16

### 3.3 Analysis of countries

**Figure 4:**
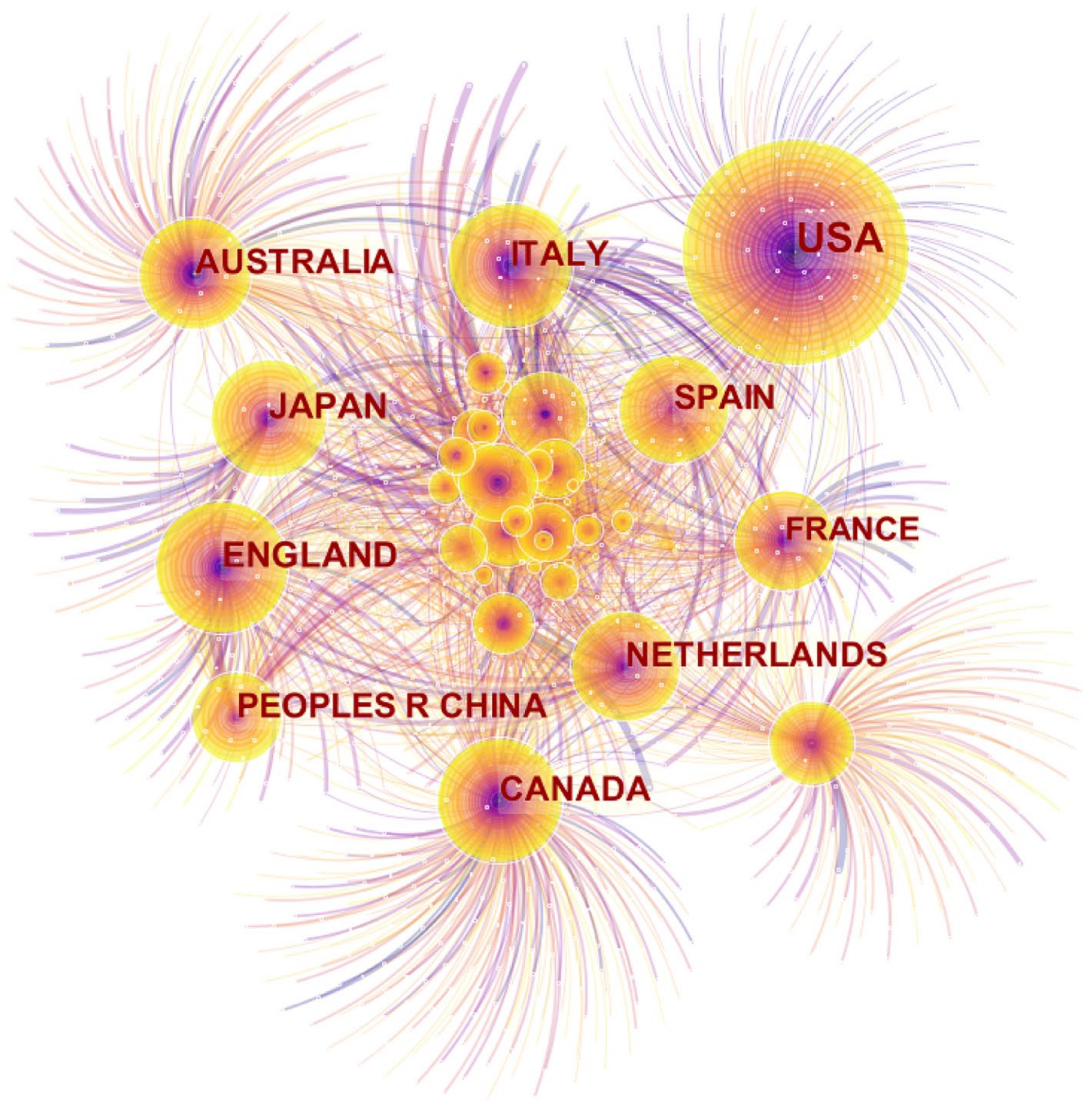
Map of countries for exercise interventions for frailty from 2003 to 2023.

Using CiteSpace, we generated a country map (Figure 4) to identify the leading countries in the domain of exercise therapies for frailty. Among the 7,093 records analyzed, publications originated from 115 countries and regions. Table 3 presents the top 10 countries in terms of publication count. The USA emerged as the foremost contributor, accounting for approximately one-fourth of the articles (1,791). England and Japan held the second and third positions, respectively.

**Table 3:** Top 10 countries/regions in the number of publications related to exercise therapies for frailty.

Country/region	Publications	Centrality
The USA	1,791	0.55
England	607	0.21
Japan	582	0.09
Spain	517	0.08
Canada	462	0.46
Italy	453	0.08
Australia	383	0.43
Netherlands	374	0.07
China	370	0.08
France	295	0.11

Figure 4 shows countries as nodes, with purple rings indicating centrality of the research. The top 3 countries with the highest centrality values are the USA (0.55), Canada (0.46), and Australia (0.43). Notably, the USA stands out with a significant difference in publication count compared to other countries, more than 1,000, and a centrality value over 0.5, indicating its dominance in terms of both quantity and importance. Over the years, exercise therapies for frailty have experienced a surge in global recognition, especially in developed countries with high levels of aging.

### 3.4 Analysis of institutional distribution

**Figure 5:**
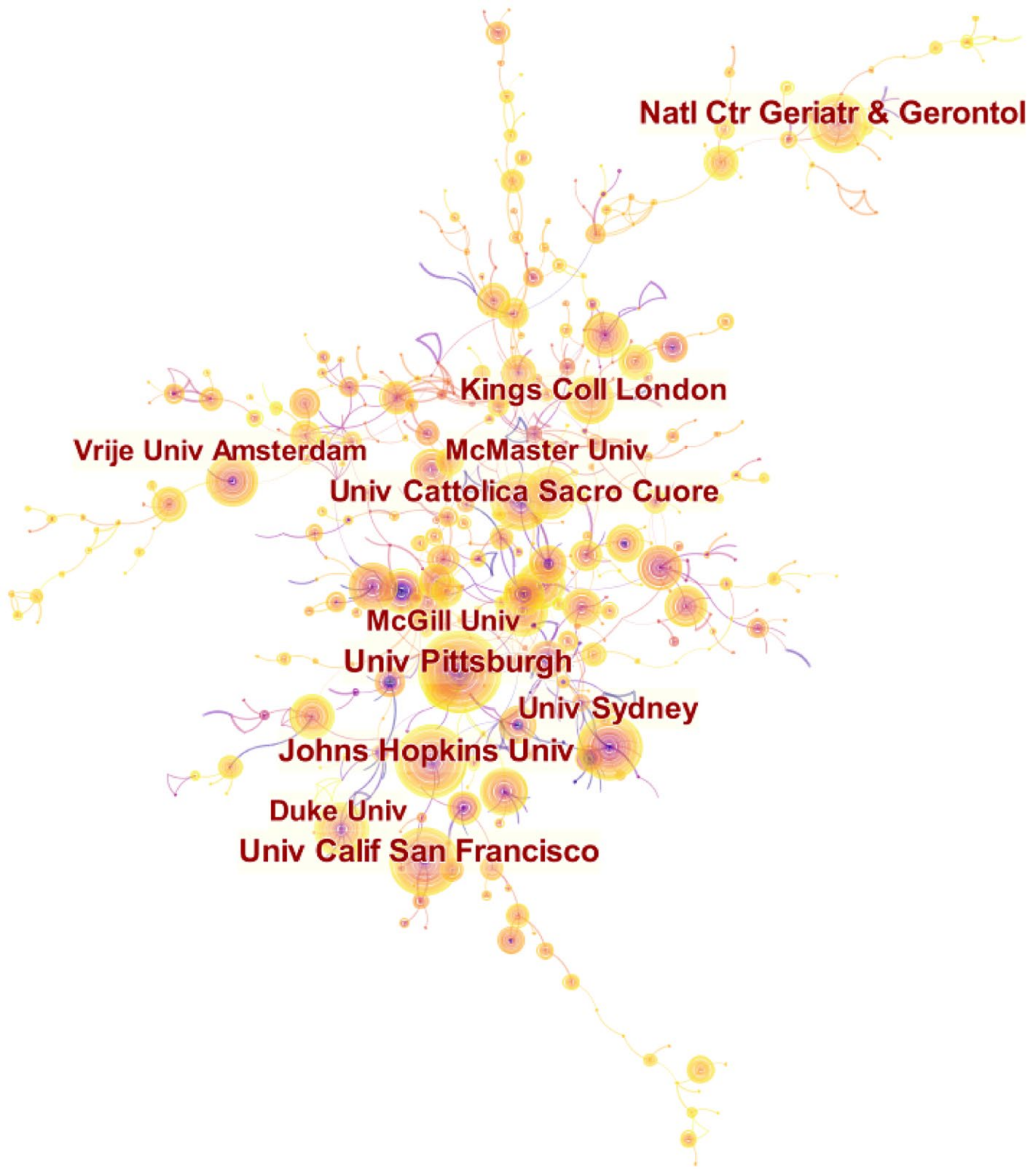
Map of institutions researching exercise interventions for frailty from 2003 to 2023.

Among the top 10 institutions with the highest prominence in the domain of exercise therapies for frailty, all except the National Center for Geriatrics and Gerontology are comprehensive universities (Figure 5). They are San Francisco, University of Pittsburgh, University of California, Johns Hopkins University, University of Sydney, Università Cattolica del Sacro Cuore, King’s College London, McMaster University, Duke University, University of Amsterdam. The only two universities with centrality >0.1 were Università Cattolica del Sacro Cuore (0.13) and University of Pittsburgh (0.22) (Table 4). The analysis of publication count and centrality reveals that the University of Pittsburgh and Università Cattolica del Sacro Cuore are the primary research institutions in this field. They constitute the core of the collaborative network. University of Pittsburgh concentrates on the topic that resistance exercise is effective for improving muscle strength and function, essential amino acid EAA (with leucine) and beta-hydroxy beta-methylbutyric acid HMB may improve muscle outcomes.

**Table 4:** Top 10 cited journals related to exercise therapies for frailty.

Institution	Publications	Centrality
University of Pittsburgh	118	0.22
University of California, San Francisco	113	0.04
Johns Hopkins University	109	0.08
The National Center for Geriatrics and Gerontology	107	0.07
University of Sydney	95	0.04
Università Cattolica del Sacro Cuore	90	0.13
King’s College London	88	0.08
McMaster University	81	0.02
Duke University	80	0.02
University of Amsterdam	80	0.09

### 3.5 Analysis of authors and referenced authors

**Figure 6:**
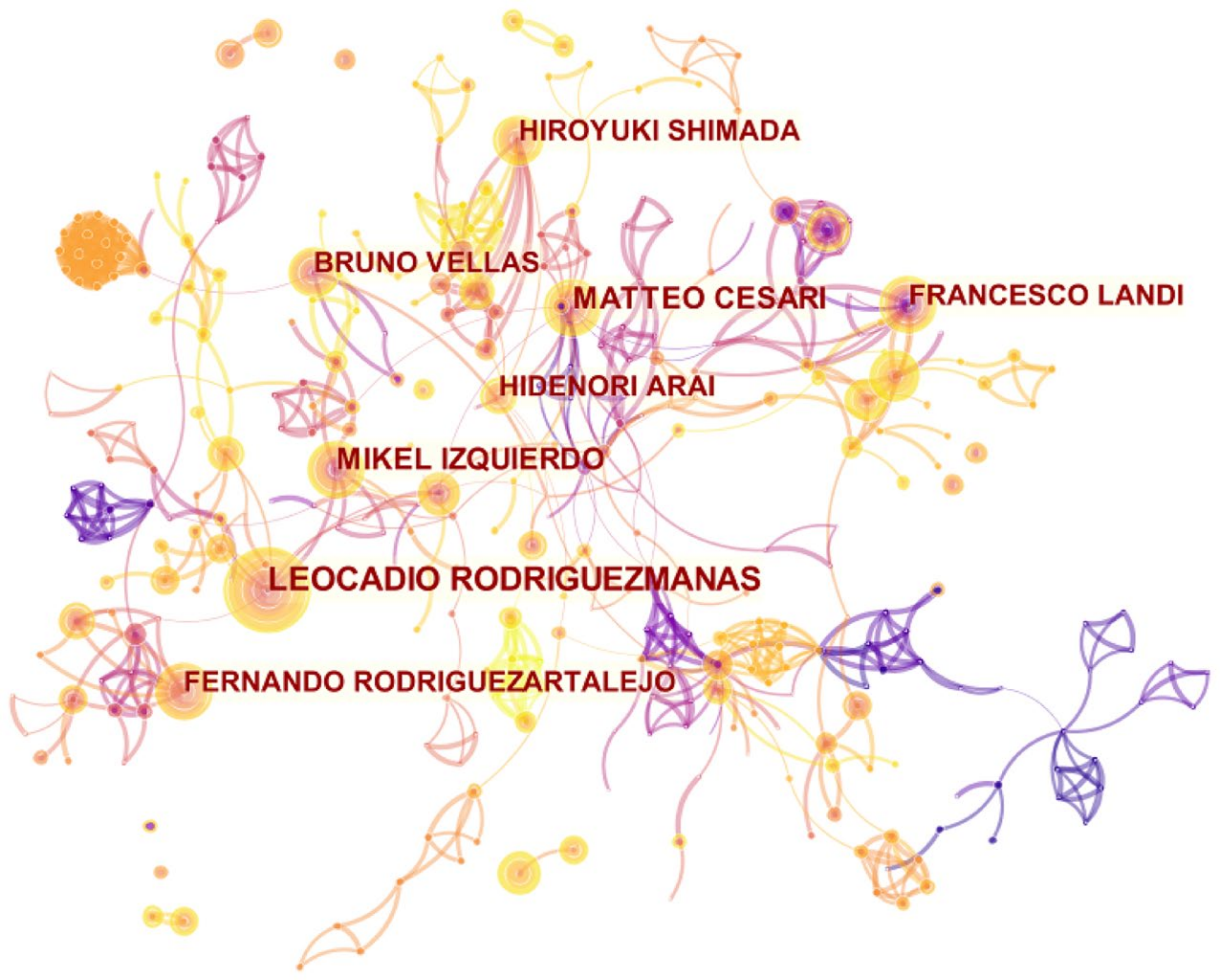
Map of authors related to exercise interventions for frailty from 2003 to 2023.

The analysis of authors for the 7,093 records resulted in 950 nodes and 1,601 links (Figure 6), showing the involvement of 33,646 authors in the publication of these articles. The top 10 authors were Leocadio Rodriguez-Manas (9), Matteo Cesari (10), Mikel Izquierdo (11), Fernando Rodriguez-Artalejo (12), Hidenori Arai (13), Hiroyuki Shimada (14), Francesco Landi (15), Emanuele Marzetti (16), Ignacio Ara (17) and Roberto Bernabei (18) (Table 5).

**Table 5:** Top 10 authors/cited authors related to exercise therapies for frailty.

Authors	Publications	Centrality	Cited authors	Frequency	Centrality
Leocadio R	65	0.05	Fried Lp	2,917	0.93
Matteo C	44	0.11	Rockwood K	1,206	0.63
Mikel I	39	0.02	Morley Je	1,088	0
Hiroyuki S	36	0.01	Guralnik Jm	918	0.4
Fernando R	35	0	Cruz-Jentoft Aj	824	0.06
Francesco L	34	0.01	Folstein Mf	743	0.12
Bruno V	32	0.03	Clegg A	735	0.12
Hidenori A	32	0.06	Cesari M	675	0.04
Emanuele M	31	0.01	Gill Tm	618	0.14
Ignacio A	28	0.02	Ferrucci L	478	0.52

Leocadio Rodriguez-Manas mainly focuses on the link between frailty, sarcopenia and diabetes (19–22), the effects of exercise intervention (23–26). Matieo Cesari not only focused on the assessment, prevention and intervention of physical performance in the old adults (27–29), but also discussed possible biomarkers in frailty, sarcopenia and other aging diseases (30–33). Mikel Izquierdo has the same focus with Leocadio Rodriguezmanas, that is the effects of exercise intervention in the old adults (24, 34, 35). Fernando Rodriguez-Artalejo focuses on the impact of different lifestyles on frailty of the old adults (36, 37). Hidenori Arai mainly focuses on some important review or consensus of frailty and sarcopenia (18, 38).

**Figure 7:**
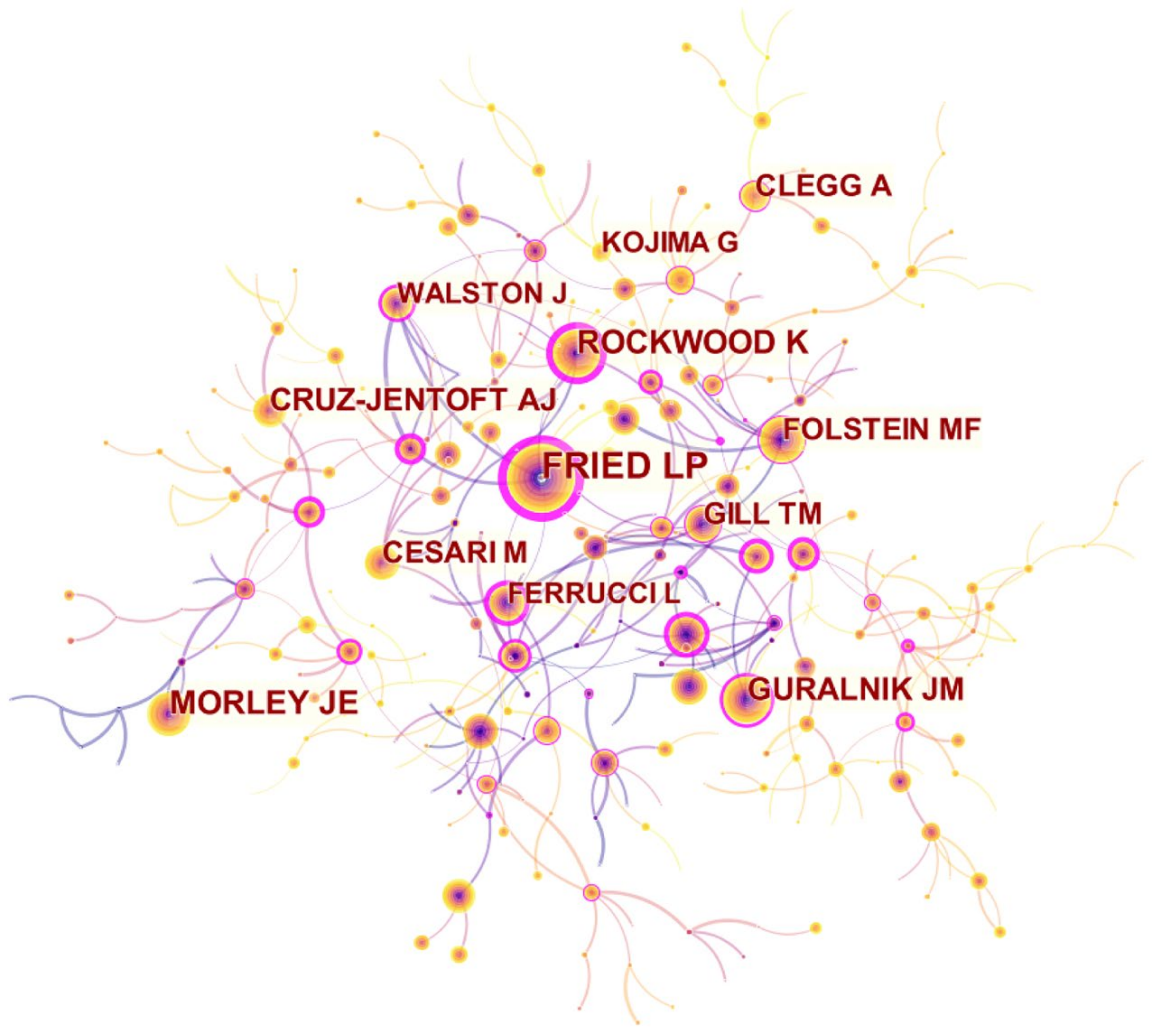
Map of cited authors related to exercise interventions for frailty from 2003 to 2023.

Figure 6 shows the visualization of referenced authors on the map. The publications of Fried LP recorded the highest citation counts (3068), followed by Rockwood K, Anonymous, Morley JE and Guralnik JM (Table 5). Figure 7 illustrates that the majority of cited authors, as indicated by the presence of purple rings, have a centrality value greater than 0.1.

### 3.6 Analysis of cited references

**Figure 8:**
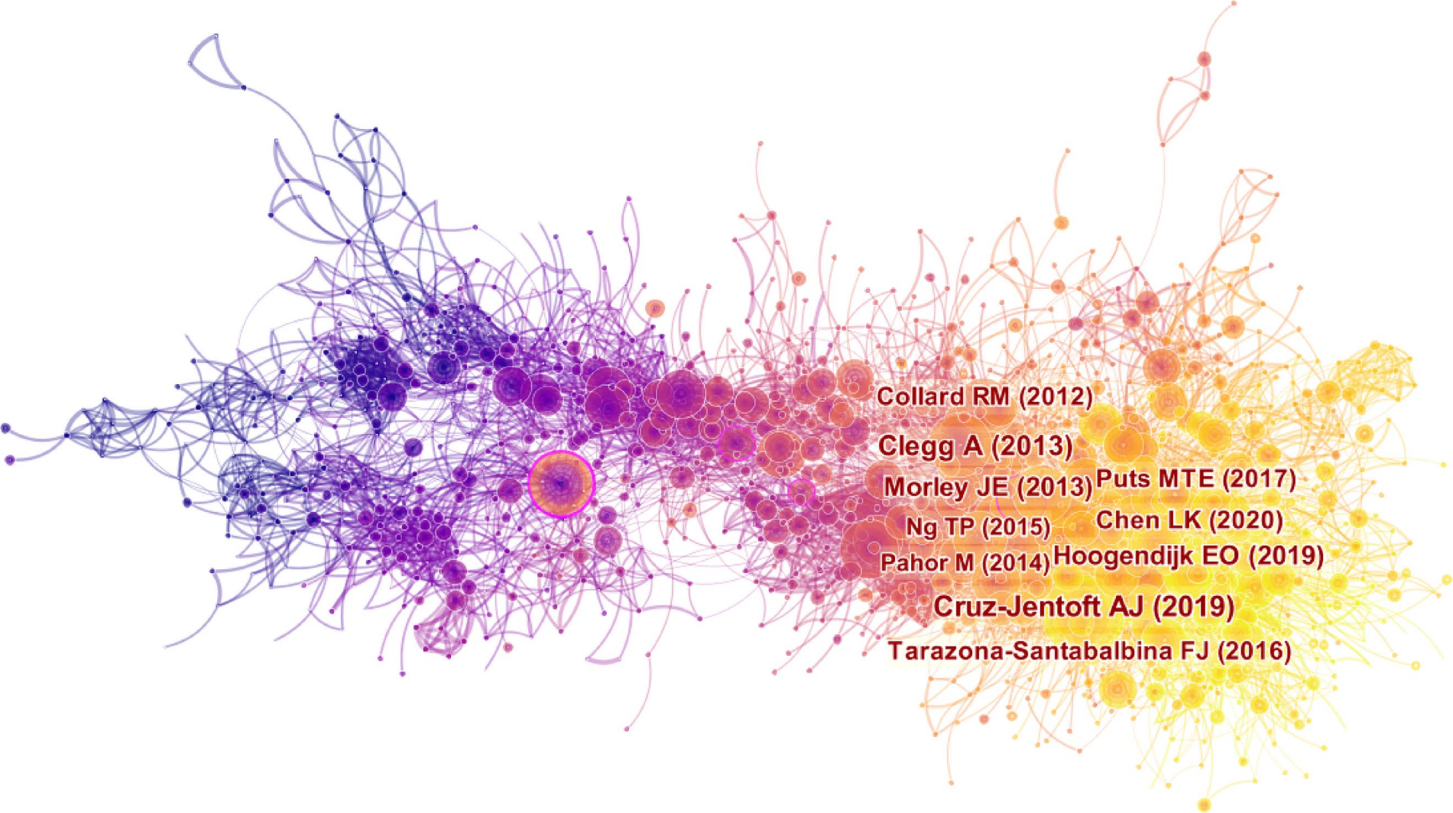
Map of cited references for exercise interventions for frailty from 2003 to 2023.

Figure 8 shows the co-citation map of referenced sources, consisting of 8,117 links and 1,617 nodes. The top frequently cited reference, published by Cruz-Jentoft AJ in 2019, holds a prominent position in the network (28). This article provides comprehensive information about the updated consensus on the diagnosis and definition of sarcopenia by the European Working Group on Sarcopenia in Older People 2 (EWGSOP2) in 2018. The second cited reference detail pathophysiology, epidemiology, instrumentation, interventions of frailty and the fourth cited reference provides a comprehensive examination of the global impact and burden of frailty, the clinical applicability of the frailty concept, potential targets for frailty prevention, and future research directions, both of them was published in Lancet in 2013 and 2019, respectively, (17, 39). The third cited reference was the 4 key consensus points on a specific type of frailty: physical frailty in 2013 (1). The fifth cited reference was systematically compared and pooled the prevalence of frailty (40). Currently, there is an absence of consensus on a universal definition and diagnostic criteria for frailty, which are subject to continuous updates and revisions. Nevertheless, these working groups offer valuable insights into the definition, prevalence, diagnosis, models, and impact of frailty. Their findings serve as valuable references for researchers involved in studying frailty.

The most cited reference, published by Puts MTE in 2017 with a centrality of 0.05, performed a comprehensive review of international policies and interventions aimed at preventing or reducing frailty among old adults living in the community (16). This scoping review revealed that only 9 out of the 14 studies demonstrated efficacy in preventing or reducing frailty. Effective interventions, such as exercise, nutrition, cognitive training, geriatric assessment and management, and pre-rehabilitation, demonstrated feasibility with adherence rates of approximately 70% in most studies. These interventions proved successful in reducing frailty levels among individuals categorized as pre-frail or frail. Table 6 presents the top 10 cited references related to exercise therapies for frailty.

**Table 6:** Top 10 cited references related to exercise therapies for frailty.

Cited authors	Frequency	Centrality
Cruz-Jentoft AJ et al.	301	0.01
Clegg A et al.	222	0.04
Morley JE et al.	195	0.04
Hoogendijk EO et al.	168	0.01
Collard RM et al.	121	0.01
Chen LK et al.	116	0.01
Puts MTE et al.	111	0.05
Tarazona-Santabalbina FJ et al.	107	0.02
Ng TP et al.	101	0.03
Pahor M et al.	100	0.01

**Figure 9:**
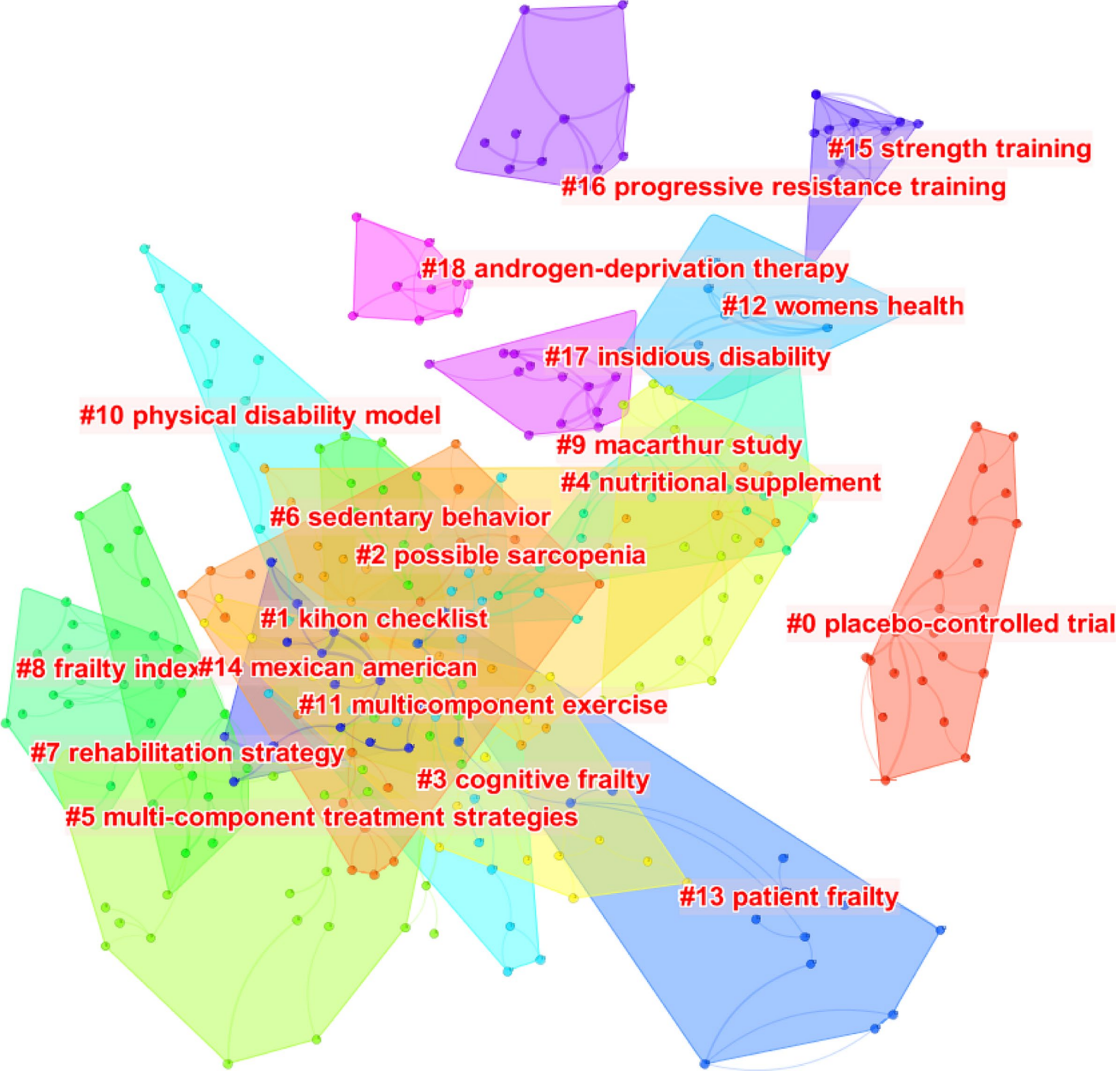
The clustering map of cited references related to exercise interventions for frailty from 2003 to 2023.

Figure 9 illustrates the results of the cluster analysis performed on the cited references, revealing the temporal distribution and topic of these references. The clustering map demonstrates a *Q* value of 0.8777 and an S value of 0.9672, showing a reliable clustering effect and high credibility. Each colored region within the clustering map illustrates a unique topic. The predominant topic in the cited references is centered around “#0 placebo-controlled trial,” “#1 kihon checklist,” “#2 possible sarcopenia,” “#3 cognitive frailty,” “#4 nutritional supplement,” “#5 multi-component treatment strategies,” “#6 sedentary behavior,” “#7 rehabilitation strategy,” “#8 frailty index,” “#9 macarthur study,” “#10 physical disability model,” “#11 multicomponent exercise,” “#12 women’s health,” “#13 patient frailty,” “#14 mexican american,” “#15 strength training,” “#16 progressive resistance training,” “#17 insidious disability,” and “#18 androgen-deprivation therapy.”

### 3.7 Analysis of keywords

**Figure 10:**
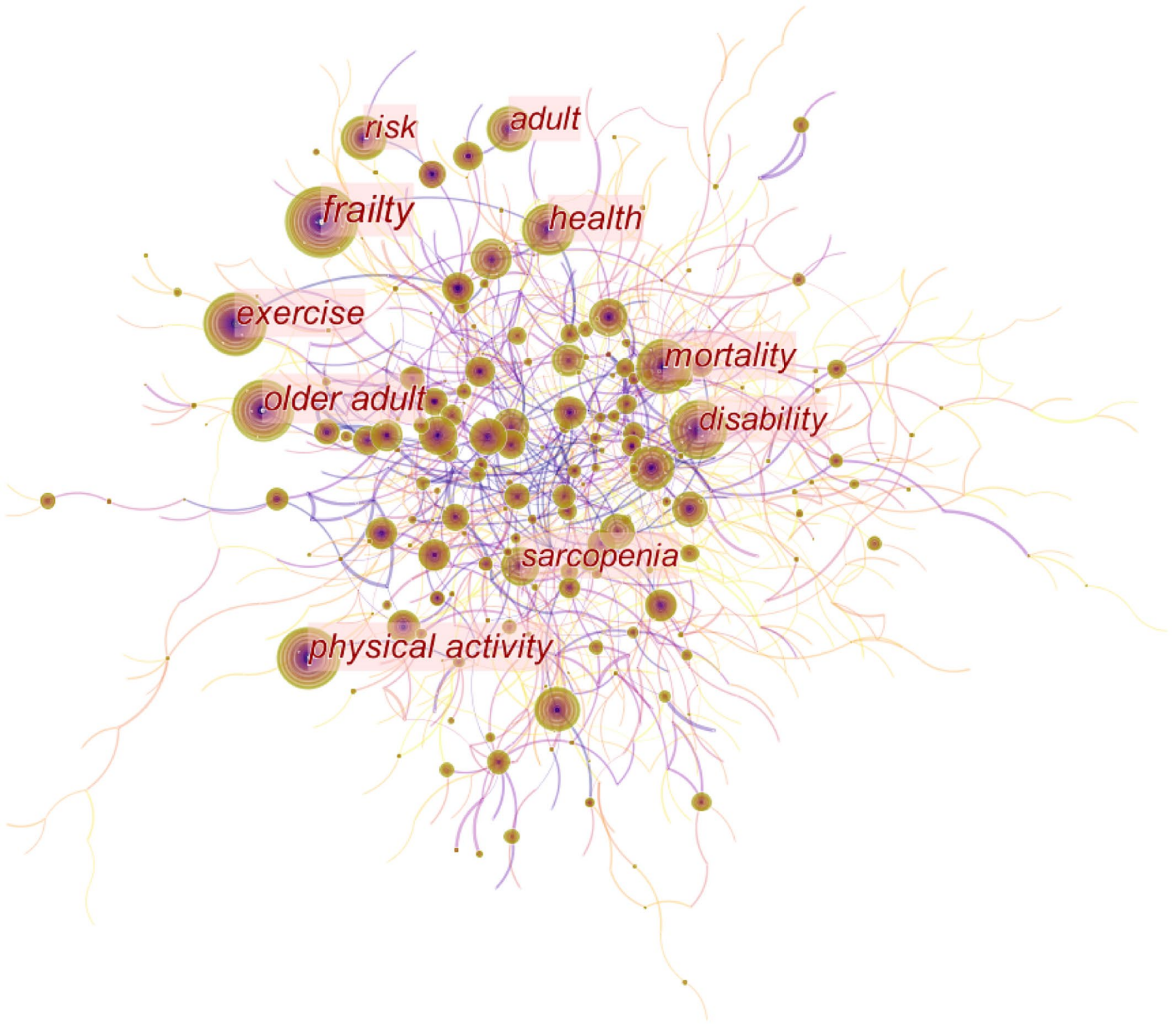
Map of keywords to exercise interventions for frailty from 2003 to 2023.

To identify hot and frontier topics, we examined the frequency and burst keywords in the domain of exercise therapies for frailty. Figure 10 shows the network map of keywords, consisting of 871 nodes and 1,253 links. This analysis uncovered a comprehensive list of 871 research keywords, providing insights into the current hot topics in the field. Based on the centrality and frequency analysis of keywords (Table 7), it is evident that the prominent keywords in terms of hot topics include “frailty,” “older adult,” “physical activity,” “exercise,” “mortality,” “health,” “disability,” “sarcopenia,” “adult,” and “risk,” while “sarcopenia” has a high centrality. The old adult is the target of attention in the field of frailty. Hence, the primary objective of interventions is to enhance physical activity among old adults, aiming to reduce risks, disabilities, and mortality. Exercise stands as the widely employed, easily accessible, and efficacious approach to address frailty. Exercise is extensively utilized in clinical settings and exerts a significant impact on health.

**Table 7:** Top 10 keywords related to exercise therapies for frailty.

Keywords	Count	Centrality	Year
Frailty	3,122	0	2003
Older adult	1,790	0	2003
Physical activity	1,357	0	2003
Exercise	1,338	0	2003
Mortality	1,242	0	2003
Health	1,116	0.01	2003
Disability	949	0.02	2003
Sarcopenia	894	0.04	2003
Adult	871	0	2003
Risk	807	0	2003

**Figure 11:**
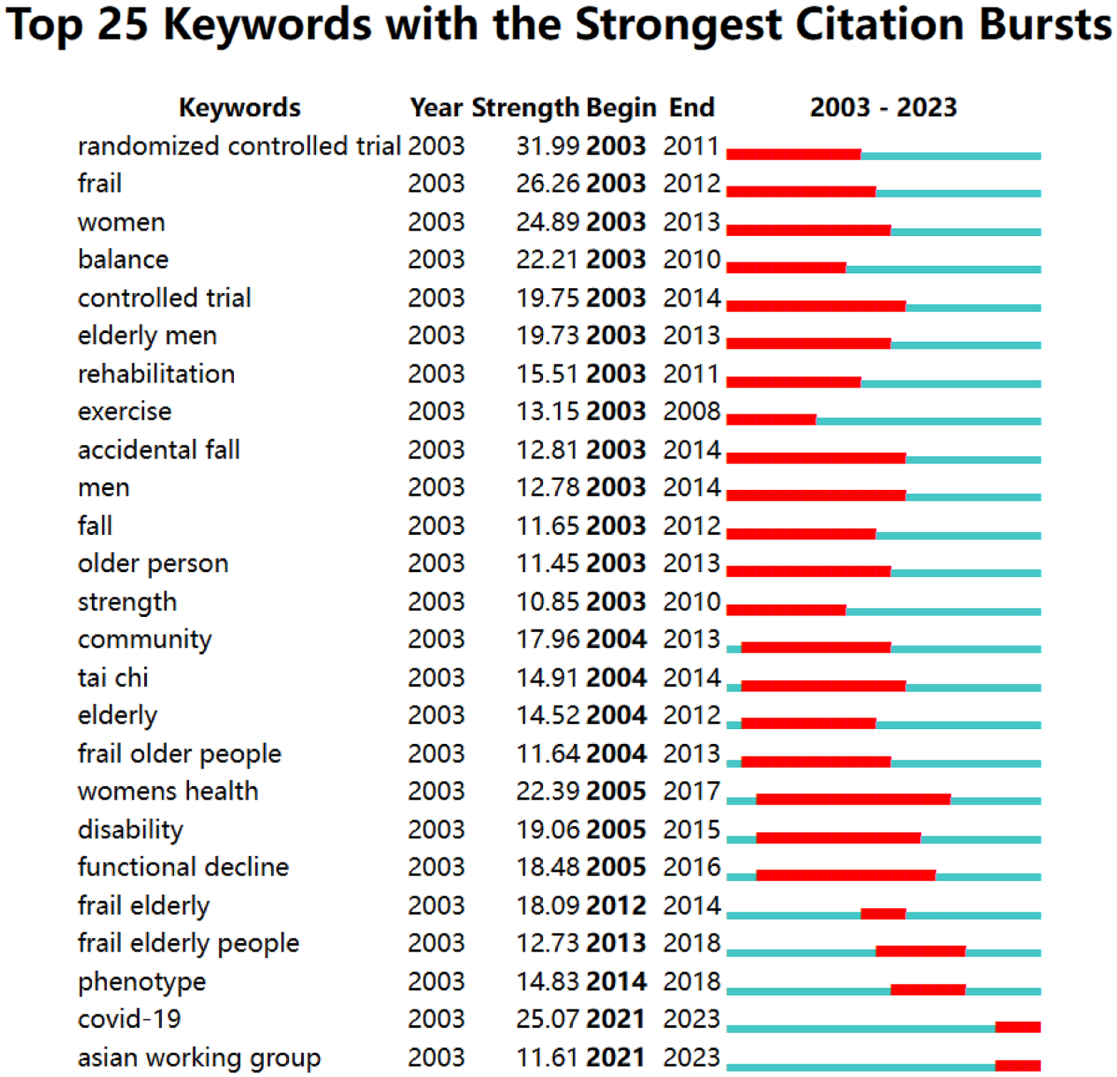
Top 25 keywords with the strongest citation burst.

Applying time series burst detection on frequently cited keywords, Figure 11 presents the identification of the top 25 keywords exhibiting significant bursts. “Randomized controlled trial,” “frail,” “women,” “balance,” “controlled trial” are subjects of frailty, “elderly men,” “rehabilitation,” “exercise,” “accidental fall,” “men,” “fall,” “older person,” “strength,” “community,” “tai chi,” “elderly,” “frail older people” emerge as key research focuses among these keywords. The keyword “randomized controlled trial” experienced a significant citation burst of 31.99 since 2003. The second most prominent keyword was “frail” with a burst strength of 26.26.

The latest burst keywords identified were “COVID-19” and “Asian working group.” The study included “COVID-19” due to the relevance of frailty as a potential prognostic indicator in COVID-19 patients. It aimed to examine the interaction between frailty and age in the old adults COVID-19 ICU patients. The “Asian working group” mentioned refers to the Asian Working Group for Sarcopenia. In 2019, they published the latest expert consensus on diagnosing and treating sarcopenia (41), emphasizing the importance of tailored diagnostic strategies for different levels of medical institutions.

**Figure 12:**
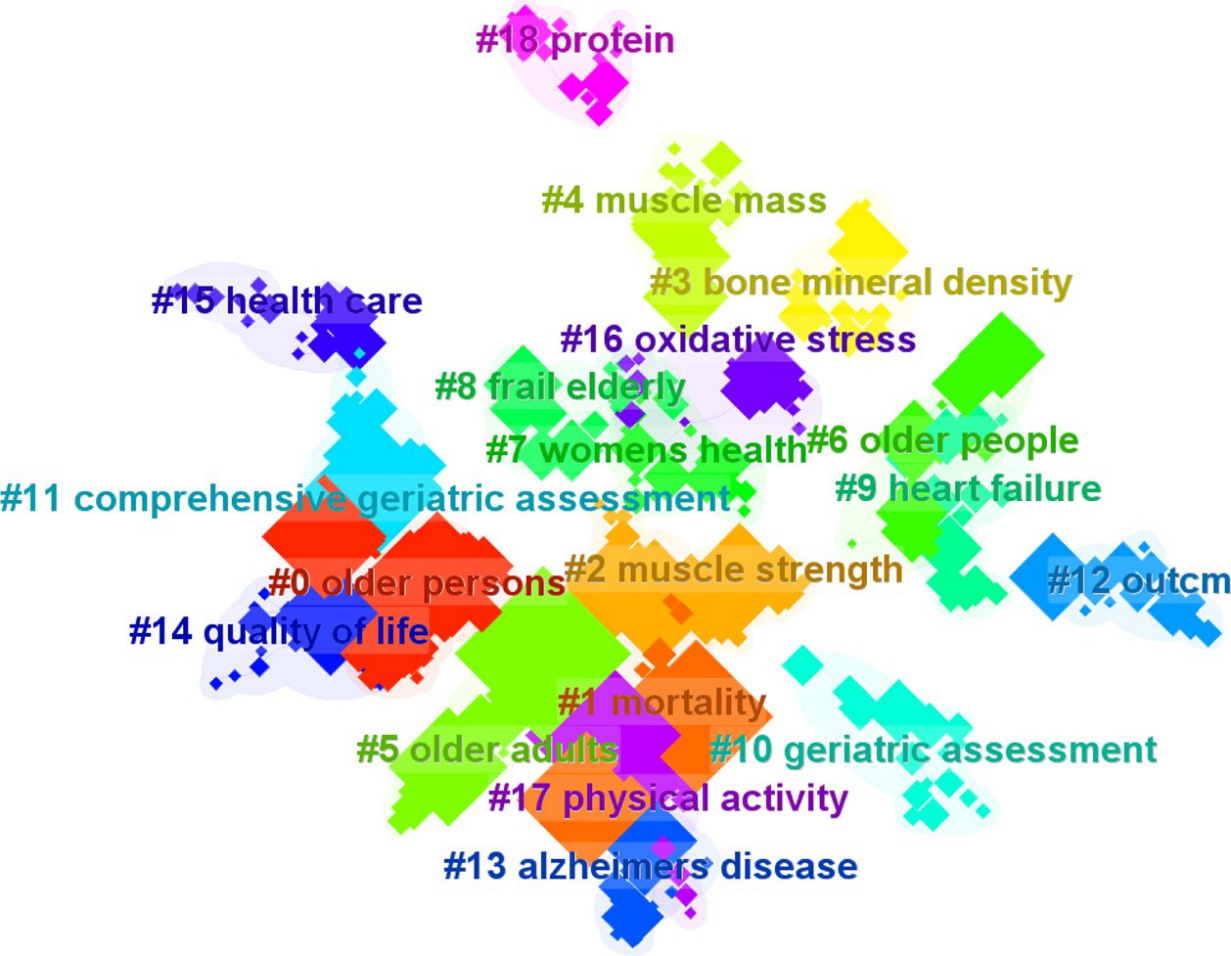
The clustering map of keywords related to exercise interventions for frailty.

**Figure 13:**
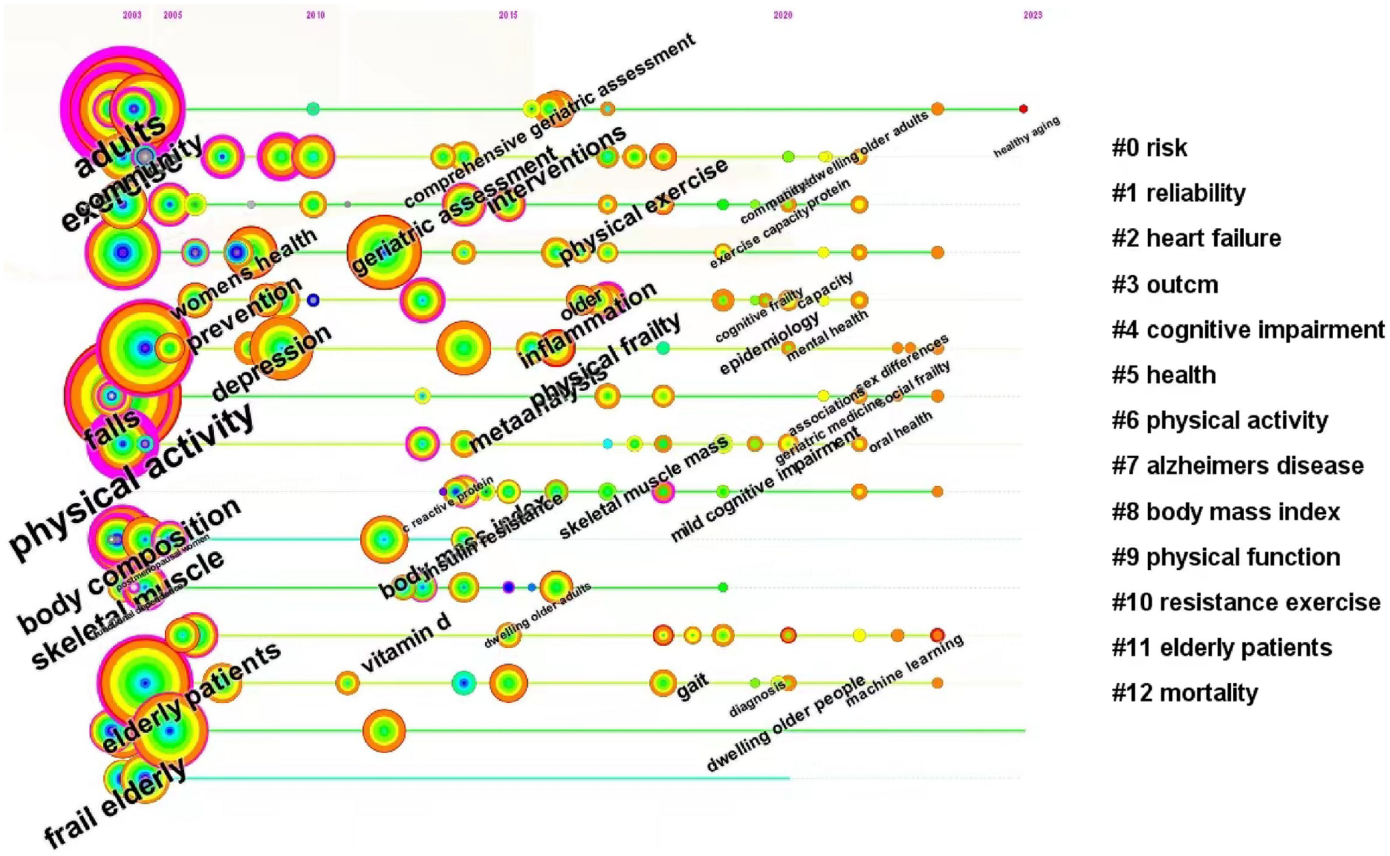
The timeline view of keywords related to exercise interventions for frailty.

Cluster analysis and summarization of these keywords offer a comprehensive overview of the present research areas concerning frailty (Figure 12). The cluster analysis produced a Q-value of 0.8492 and an S-value of 0.9575, confirming the validity and significance of the clustering results. Nineteen clusters were formed to represent the prevailing trends in current research, including “#0 older persons,” “#1 mortality,” “#2 muscle strength,” “#3 bone mineral density,” “#4 muscle mass,” “#5 older adults,” “#6 older people,” “#7 women’s health,” “#8 frail elderly,” “#9 heart failure,” “#10 geriatric assessment,” “#11 comprehensive geriatric assessment,” “#12 outcm,” “#13 alzheimers disease,” “#14 quality of life,” “#15 health care,” “#16 oxidative stress,” “#17 physical activity,” and “#18 protein.” From the timeline view (Figure 13), “#0 risk” and “#11 elderly patients” are still hot topics, and the other clusters had varying degrees of heat loss, especially for “#7 alzheimers disease.”

## 4 Discussion

### 4.1 Exercise therapies for frailty: general knowledge structure

Exercise plays a crucial role in the rehabilitation of frailty and is a prominent research focus. Exercise rehabilitation provides the benefits of simplicity and personalized exercise prescriptions. This research retrieved 7,093 records on exercise therapies for frailty from the WoSCC, spanning from 1st January 2003 to 31st August 2023. Through bibliometric analysis using CiteSpace, we identified the spatial and temporal distribution as well as the research hotspots in the domain of exercise therapies for frailty. In the last two decades, there has been a significant surge in publications concerning exercise therapies for frailty. In this research, the journal BMC Geriatrics published the highest number of articles (246), while the journal J Gerontol A-Biol received the highest number of citations (4,774). Countries and institutions conducting research on exercise therapies for frailty exhibited strong collaboration. Notably, the USA, England, Japan, Canada, Australia, China, and several European countries emerged as leading contributors in terms of publication centrality and rate. These developed countries played a significant role in advancing the domain of exercise therapies for frailty. The University of Pittsburgh was identified as the most productive institution in the domain of exercise therapies for frailty. Numerous authors from various countries have contributed to studies within this research area. Among the authors, Leocadio Rodriguez-Manas emerged as the most productive author, while Fried Lp was the top-cited author. The most centrally cited reference was a consensus paper published by Cruz-Jentoft AJ in 2019, which focused on the definition and diagnosis of sarcopenia by the EWGSOP2 in 2018. Cluster analysis of the cited reference reveals that the latest cited hot topics were “kihon checklist,” “possible sarcopenia,” “cognitive frailty,” “nutritional supplement,” “multi-component treatment strategies,” “sedentary behavior,” “rehabilitation strategy,” “frailty index,” “macarthur study,” “physical disability model,” and “multicomponent exercise,” respectively.

The prominent keywords in the domain of exercise therapies for frailty include “frailty,” “older adult,” “physical activity,” “exercise,” “mortality,” “health,” “disability,” “sarcopenia,” “adult,” and “risk.” The keyword “randomized controlled trial” exhibited the most significant citation burst, while the second keyword with a burst was “frail.” A total of 18 clusters were identified, and the top 10 clusters were related to “older persons,” “mortality,” “muscle strength,” “bone mineral density,” “muscle mass,” “older adults,” “older people,” “women’s health,” “frail elderly,” and “heart failure.” Keywords such as “women,” “balance,” “controlled trial,” “elderly men,” “rehabilitation,” “exercise,” “accidental fall,” “men,” “fall,” “older person,” “strength,” “community,” and “tai chi” indicate the targets or mechanisms of exercise therapies for frailty.

### 4.2 Exercise for frailty rehabilitation of vital human body systems

Exercise therapy is one of the effective methods to prevent and treat frailty. Proper exercise usually slows down aging in vital human body systems, which include the muscular system, skeletal system, cardiopulmonary system, nervous system, and endocrine metabolic system etc. The keyword information related to this study shows that the muscular system is the body system with the highest centrality.

### 4.3 Exercise for frailty of musculoskeletal system

Frailty of the musculoskeletal system is most commonly characterized by osteoporosis and sarcopenia, and exercise is one of the very important interventions to improve osteoporosis and sarcopenia.

Frailty can directly or indirectly lead to reduced bone density, decreased skeletal muscle mass and strength, and consequently increased risk of fracture (15). It has been shown that the mechanical stress generated by physical activity increases muscle and bone mass and osteoblast activity (14, 42). However, not all forms of exercise are equally osteogenic. Progressive resistance exercise or in combination with other interventions (protein, calcium and vitamin D supplementation) is optimal for improving muscle and bone density in old adults (12, 13).

Characteristics of frailty and sarcopenia are closely related: weight loss, fatigue, reduced grip strength, slowed gait speed, and reduced physical activity (11). There is a large number of evidence from systematic reviews and meta-analyses that resistance exercise significantly improves muscle mass, strength, and balance function in patients with sarcopenia (43–47).

### 4.4 Exercise for frailty of cardiopulmonary system

Cardiorespiratory system frailty can cause chronic obstructive pulmonary disease (COPD), chronic heart failure (HF), and other diseases, and can increase susceptibility to COVID-19 in old adults. Exercise is safe and convenient compared to medication and is an effective measure to improve a frail cardiopulmonary system.

Frailty decreases left ventricular ejection fraction and affects peak oxygen uptake (10). High-intensity interval training (HIIT) has been shown to have significant efficacy in improving cardiovascular outcomes and frailty in old adults (48). In patients with COPD, common symptoms include quadriceps atrophy, decreased physical fitness, and reduced pulmonary function, all of which are associated with frailty (49). However, long-term (≥5 months) multi-component exercise intervention has a positive effect on improving cardiorespiratory fitness and activities of daily living in patients (50). In clinical practice, frailty interferes with the implementation of strategies to guideline-directed medical therapy, leads to poor prognosis, and reduces physical and cognitive function in patients with HF with reduced ejection fraction (51, 52). Furthermore, early identification and intervention of frailty in chronic kidney disease (CKD) and HF patients are priorities to improve their quality of life (53). In addition, for nutritional strategies, appropriate protein intake may improve frailty in non-dialysis CKD patients (54). Biomarkers of frailty (such as elevated procalcitonin, transferrin, cortisol, C-reactive protein, interleukin-6, lactate dehydrogenase, as well as low vitamin D levels), predict the severity of COVID-19 in old patients (55), and frailty also reduces susceptibility and immunity to COVID-19 in old adults, and, conversely, COVID-19 makes patients even more frail, thus creating a vicious cycle (56). Chen et al. (57) implemented the Otago exercise plan intervention program during the COVID-19 pandemic to improve physical function in cognitive frailty old adults, reduce the harms of prolonged sedentary behaviors, and reduce depressive symptoms and improve mental health.

### 4.5 Exercise for frailty of nervous system

The most common clinical diseases of nervous system frailty are Parkinson’s disease, Alzheimer’s disease and depression in aging.

To our knowledge, exercise has a positive effect on slowing the progression of Parkinson’s disease. It has been shown that open-space activities, aerobic exercise, and moderate-intensity exercise reduce neuroinflammation and improve the quality of life in patients with Parkinson’s disease (58, 59). Additionally, exercise increases neuroplasticity through activation of brain neurotrophic factors, thereby improving cognition (60).

Frailty may affect the diseases progression through the following pathways: firstly, frailty leads to loss of muscle strength and flexibility, resulting in reduced ability to perform activities of daily living, such as dressing and bathing, in old adults; secondly, frailty also increases the risk of fractures and falls, further limiting their independence and decreasing their quality of life; furthermore, frailty may be related to cognitive decline and behavioral problems in Alzheimer’s patients. One systematic review and meta-analysis provided sufficient evidence to suggest that old adults over 65 years of age should adhere to regular physical activity to maintain health and strength, and that old adults in pre-frailty or frailty, in particular, should be strengthened with cognitive function assessment and interventions (61).

### 4.6 Exercise for frailty of endocrine metabolic system

The most common clinical diseases of the endocrine and metabolic system frailty are diabetes and obesity, and the relationship between diabetes and obesity and frailty is interactive. The interactions among diabetes, obesity, and neurodegenerative diseases are complex and multifaceted. Obesity is a major risk factor for developing type 2 diabetes, and diabetes, in turn, increases the risk of neurodegenerative diseases. Chronic inflammation, oxidative stress, impaired insulin signaling, and dysregulated glucose and lipid metabolism are shared pathophysiological mechanisms (62, 63). These conditions have mutual influences, with obesity and diabetes exacerbating each other and both contributing to the progression of neurodegenerative diseases. And appropriate aerobic exercise and resistance exercise can prevent and treat diabetes and obesity, improving muscle function, mental health, and quality of life (64, 65).

### 4.7 Possible pathophysiological mechanisms of exercise for frailty

Exercise plays a crucial role in improving frailty through various pathophysiological mechanisms. Regular exercise, particularly resistance training, enhances muscle strength and mass, counteracting the age-related decline in muscle tissue (9). It also improves metabolic function by enhancing glucose utilization, insulin sensitivity, and lipid metabolism, while exerting anti-inflammatory effects, modulating immune function, and reducing systemic inflammation (66). Additionally, exercise reduces oxidative stress, improves mitochondrial function, and promotes cellular health (67). Moreover, exercise promotes neuroplasticity, enhances cardiovascular fitness, increases bone density and strength, and regulates hormonal balance (15). These multifaceted mechanisms collectively contribute to the overall improvement of frailty, along with the positive psychological effects of exercise interventions, including improved mood, reduced depression and anxiety symptoms, enhanced well-being, and increased self-esteem and social interaction (68). However, it is crucial to consider individual capabilities and seek medical guidance when designing exercise programs for frail individuals.

### 4.8 Future trends

From the timeline Figure 13, it can be concluded that the clusters with the topics of “#9 physical function” and “#10 resistance exercise” have seen a gradual decrease in research activity and attention after 2014 and 2018, respectively, indicating a decrease in the relevance of each keyword under this cluster, an increase in research diversity, an increase in interdisciplinary cooperation or cross-study, and a segmentation of research directions, and that only the clusters with the topics of “#0 risk” and “#11 elderly patients” have had a continuous research hot spots up to now, suggesting that it is a hotspot of research in this field, and that, furthermore, the greatest number of articles and the highest quality of research have been published in the topics of the various clusters before the year 20.

With increasing aging and the popularity of exercise for health, exercise therapies have shown promising applications in frailty rehabilitation, and researchers are increasingly focusing on the direction of exercise therapies in the early stages of frailty. HF and higher cerebral dysfunction are common early clinical symptoms of frailty, which in turn increases the risk of cardiovascular and cerebrovascular diseases. Therefore, active intervention in the early phases of frailty can reduce the incidence of cardiovascular disease, of which exercise intervention is a cost-effective and practical modality. The analyses of the timeline perspective suggest that cognitive impairment, HF and Alzheimer’s disease are closely associated with exercise intervention for prefrailty, which provides a research direction for studying exercise intervention for cardiovascular and cerebrovascular diseases in the early stages of prefrailty.

### 4.9 Strengths and limitations

This research demonstrates a valuable overview of the hotspots and study frontiers in the domain of frailty, offering meaningful insights for further investigations. However, it is important to acknowledge some limitations. Firstly, the analysis is restricted to the data from the WoSCC, excluding other databases like PubMed or Embase. Secondly, the study focuses on English-language literature, potentially excluding valuable research from non-English-speaking countries. Lastly, it should be noted that the number of centrality and citations may vary depending on the time period of the search, and thus, this study specifically reflects the study frontiers within the past 20 years.

## 5 Conclusion

This research employs CiteSpace, a visualization software, to uncover potential collaborators, hot topics, partner institutions, and emerging perspectives in exercise for frailty research. Through bibliometric analysis, exercise emerges as the most significant and crucial intervention in frailty rehabilitation. The search for scientifically sound, effective, and safe exercise prescriptions remains a focal point for future study. Key strategies to intercept frailty in daily clinical practice include: screening for frailty, comprehensive patient assessment, multidisciplinary collaboration, development of individualized care plans, health promotion, medication evaluation, follow-up and monitoring, and patient and caregiver education. Through these strategies, signs of frailty can be detected early and intervene accordingly to improve patient prognosis and quality of life. In summary, our research offers a comprehensive overview of the development and key aspects of exercise therapies for frailty in the past two decades, providing valuable guidance for further exploration and advancement in this field.

## Data availability statement

The original contributions presented in the study are included in the article/supplementary material, further inquiries can be directed to the corresponding authors.

## Author contributions

WX: Data curation, Software, Visualization, Writing – original draft. XZ: Conceptualization, Formal analysis, Investigation, Writing – original draft. MZe: Data curation, Formal analysis, Methodology, Writing – original draft. SW: Project administration, Supervision, Writing – original draft. YH: Methodology, Project administration, Writing – review & editing. MZh: Project administration, Resources, Supervision, Visualization, Writing – review & editing.
